# A spot-matching method using cumulative frequency matrix in 2D gel images

**DOI:** 10.1080/13102818.2014.949036

**Published:** 2014-11-28

**Authors:** Chan-Myeong Han, Joon-Ho Park, Chu-Seok Chang, Myung-Chun Ryoo

**Affiliations:** ^a^ Department of Computer Engineering, Yeungnam University, Gyeongsan, Gyeongbuk, Korea; ^b^ Department of Computer Engineering, Kyungwoon University, Gumi-si, Gyeongbuk, Korea

**Keywords:** spot matching, neighbour spot, cumulative frequency matrix, topological pattern, matching probability

## Abstract

A new method for spot matching in two-dimensional gel electrophoresis images using a cumulative frequency matrix is proposed. The method improves on the weak points of the previous method called ‘spot matching by topological patterns of neighbour spots’. It accumulates the frequencies of neighbour spot pairs produced through the entire matching process and determines spot pairs one by one in order of higher frequency. Spot matching by frequencies of neighbour spot pairs shows a fairly better performance. However, it can give researchers a hint for whether the matching results can be trustworthy or not, which can save researchers a lot of effort for verification of the results.

## Introduction

Researchers in the biological field have a need for automated data analysis techniques to detect and recognize differences in the patterns of proteins on two-dimensional electrophoresis (2DE). Two-dimensional polyacrylamide gel electrophoresis (2D PAGE) is a process that can detect thousands of polypeptides, separating them by apparent molecular weight and isoelectric point (PI). It thus provides a more realistic and global view of cellular genetic expression than any other technique.[1]

When analysing images from 2D gels, there is a reference image that represents the distribution of a sample of proteins in reference conditions (normal or healthy status). In such case, molecules are labelled and their spatial location is known. Test images are then presented. In the case of test images, the spatial location of the proteins is unknown. Usually, a comparison between a test image and the reference image is performed in order to establish the correspondence between proteins, which is called ‘spot matching’. Subsequently, both images are compared in order to establish a diagnosis based on the differences in the pattern of the identified proteins.[2]

This paper proposes a complementary method to the previous method called ‘spot-matching method by topological patterns of neighbour spots’ (TPNS).[3] TPNS is a very creative method but it shows poorer performance as the number of spots in the reference gel and target gel increases. It is because the similar patterns of neighbour spots happen to increase. This paper presents how to verify the results from TPNS and how to check the result with the least effort, using evaluated probabilities for correct matching.

Spot matching by centroids of spots can be considered as a point pattern matching problem.[4] The typical method of spot matching in 2DE gel images is the method by landmarks which are manually defined. Spots around landmarks are matched in turn.[5,6] Piecewise bilinear mapping is obtained using manual landmarks.[7] Initial matching is performed with landmarks and subsequent matching is performed with best matching of neighbour spots.[8] Some methods enable users to check and correct the matching results.[9] The majority of conventional software programs use manually defined landmarks. Nevertheless, the process of manually defining landmarks has high error rates, for it is tedious and tiresome.

A method called ‘iterative closest point’ (ICP) is proposed in the latest studies of automated protein spot matching.[10] In ICP, matching is performed according to distances between matched pairs of spots from two sets of spots and parameters of non-linear transformation are acquired. The calculated parameters are used in transforming gels non-linearly and distances between spot pairs are recalculated and the condition of converge is tested. ICP is to repeat a series of these processes. Euclidean distance and shape context distance are used as a distance measure. It assumes that 2DE gel images are under non-linear deformation but it is actually only locally that they are under non-linear deformation.

A method based on hierarchical structure and minimization of energy is proposed.[9] The proposed algorithm for spot matching is an integration of the hierarchical-based and optimization-based methods. The hierarchical method is first used to find corresponding pairs of protein spots satisfying the local cross-correlation and overlapping constraints. The matching energy function based on local structure similarity, image similarity and spatial constraints is then formulated and optimized. There is a trial which uses a quadratic assignment formulation together with a correspondence estimation algorithm based on graph matching which takes into account the structural information between the detected spots.[11] Similarly, some studies propose matching methods motivated by the preservation of topology. To compare the similarity of topology patterns, distances and angles among neighbour spots are compared.[12]

## Materials and methods

### Spot-matching method using topological pattern of neighbour spots

This paper is very closely related to a previous report on the spot-matching method using TPNS.[3] The drawback of NPNS is that it has more false positive results as the number of spots increases. This happens because similar patterns are increased as the number of spots increases. This paper describes how to improve the accuracy of TPNS using an accumulated frequency matrix. The TPNS approach is explained briefly and a new method complementary to TPNS is proposed.

The essential part of TPNS is to match spots from the reference gel and the target gel, using the topological similarity of neighbour spots. Neighbour spots of a certain spot *p_i_* can be defined as spots whose edges are connected to *p_i_*. Edges are formed according to the graph theory. As a result, neighbour spots can be determined when a set of points are given and a certain graph theory is applied to them. Gabriel graph, Delaunay graph, relative neighbour graph and *k*-nearest neighbour graph (*k*-NNG) are frequently used graphs in the point pattern matching. The neighbour spots can be described as [3] follows:
(1) 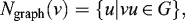
where *v* is a spot to be matched and it is called ‘central spot’ and *N*_graph_ *(v)* is a set including neighbour spots defined by graph.

**Figure 1.**
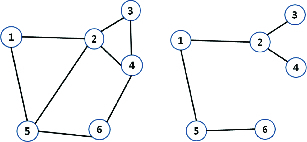
Examples of neighbour spots by Gabriel graph (left) and relative neighbour graph (right).

In Figure 1(a), there are six spots and a Gabriel graph is applied to form edges between the spots. In this case, the neighbour spots of spot 5 are spot 1, 2 and 6. This can be described as
(2) 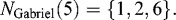

In Figure 1(b), a relative neighbour graph is applied to the exactly same set of spots as that in Figure 1(a) and the neighbour points of spot 5 are spot 1 and 6. This can be written as
(3) 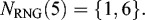



Different graph theories form different edges even for the same point pattern. The definition of neighbour spots depends on what kind of graph is used. The locations of the neighbour spots are termed ‘topological pattern’. TPNS uses the *k*-nearest neighbour graph where *k* is 5. The *k*-nearest neighbour graph is a graph in which two vertices *p* and *q* are connected by an edge if the distance between *p* and *q* is among the *k*th smallest distances from *p* to other *q* from *P*.

**Figure 2.**
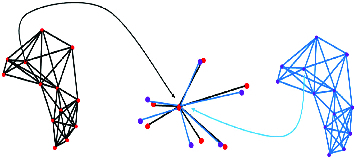
Spot matching by topological patterns of neighbour spots.

Let *P* = {*p_1_*,*p_2_*,*p_3_*,…,*p_m_*} be a point set of the reference gel and *Q* = {*q_1_*,*q_2_*,*q_3_*,…,*q_n_*} a point set of the target gel where *p_i_* = (*x_i_,y_i_*) and *q_j_* = (*x_j_,y_j_*) are the coordinates of the point in the *x*–*y* plane. TPNS estimates correspondence, using the similarity between patterns of *N*_5-NNG_(*p_i_*) and *N*_5-NNG_(*q_j_*). If central spots *p_i_* and *q_j_* from the reference gel and the target gel are given, two sets of neighbour spots, *N*_5-NNG_(*p_i_*) and *N*_5-NNG_(*q_j_*) are extracted as in Figure 2 and the similarity between the two patterns is compared to determine whether two central spots are a good match or not.

One of the two patterns must be adjusted before they are compared because scale, rotation and transposition parameters for the reference and the target gel might be different due to the image-scanning process. It does not matter which one should be transformed and the patterns from the target gel are transformed for the sake of convenience in the paper.[3] Similarity transformation is used and the central spot pair and the pivot spot pair are used to calculate similarity parameters.

The central spot pair is two spots to be matched (*p_i_,q_j_*) and the pivot spot pair is two spots, respectively, from *N*_5-NNG_(*p_i_*) and *N*_5-NNG_(*q_j_*)*_._* They must be in the relationship of matching. The problem is that the pivot spot pair cannot be known until the matching process is finished and it might be multiple. In [3], all of the possible combinations of two spots from *N*_5-NNG_(*p_i_*) and *N*_5-NNG_(*q_j_*) are considered as the pivot spot pair and all of the cases are tried to compare the topological patterns. The best pivot spot pair can be picked easily in that it definitely produces the best matching result.

**Figure 3.**
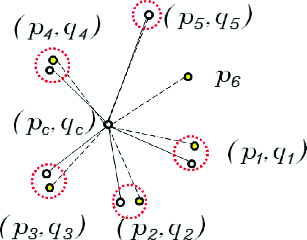
Process of matching neighbour spots.

Parameters for similarity transformation are obtained after the central spot pair and the pivot spot pair is selected and the pattern from the target gel is transformed. The transformed pattern of *N*_5-NNG_(*q_j_*) is then superimposed on the pattern of *N*_5-NNG_(*p_i_*) at the centre of the central spot (*p_i_,q_j_*) as in Figure 3. The next step is to get neighbour matched pairs which have the shortest distance between (*p_k_, q_l_*) where *p_k_∈N*_5NNG_(*p_i_*) and *q_l_∈N*_5-NNG_(*q_j_*) as in Figure 3.

Similarity can be evaluated using neighbour matched pairs. Hausdorff distance (HD) is most commonly used when obtaining the similarity of patterns; it is the ‘maximum distance of a set to the nearest point in the other set’ and is given as follows:
(4) 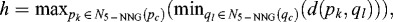
where *p_k_* and *q_l_* are spots from *N*_5-NNG_(*p_c_*) and *N*_5-NNG_(*q_c_*), respectively, and *d*(*p_k_, q_l_*) is the Euclidean distance function between these two spots. Finally, the normalized Hausdorff (NHD) distance is utilized because topological patterns are transformed by the central spot pair and the pivot spot pair, which changes the scale parameter of HD.

If there are many outlier spots, a very small value of NHD might be obtained because only a small number of matched pairs are produced. Many outlier spots mean that there is less possibility for two central spots to correspond to each other. For this reason, NHD is not a sufficient criterion of matching for two spots. Three criteria are introduced for a better matching result; the more the neighbour matched pairs, the fewer the outlier spots and the less the NHD.

### Proposed method

The problem of TPNS is that the false matching rate increases as the number of spots increases, meaning that the probability of similar topological patterns also becomes higher. The results from spot matching should be double checked in the case of densely populated spot patterns. The idea proposed here is to match the spots as many times as the number of neighbour spots is throughout the entire matching process. The matching frequency for a spot pair is equal to the number of neighbour spots if it is a correct one. Falsely matched pairs happen sporadically and the frequencies of matching for false positive pairs are relatively low.

**Figure 4.**
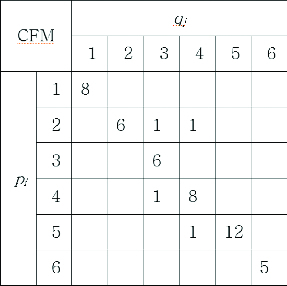
Cumulative frequency matrix.

First, a cumulative frequency matrix is used to accumulate frequencies for each neighbour spot pair, while the central spot pairs are matched. The cumulative frequency matrix can be described as in Figure 4. In this example, the reference gel consists of six spots and the target gel consists of six spots. The same spot numbers are assigned for matched spot pairs. There are six spots from the target gel on the *X*-axis of and six spots from the reference gel on the *Y*-axis. The total number of bins is 36 and each bin stores the frequency of matching for a neighbour spot pair (*p_i_, q_j_*). For example, the frequency of 12 is stored in the bin of spot pair (*p_5_, q_5_*), meaning that *p*_5_ and *q*_5_ are matched as a neighbour spot pair 12 times throughout the spot-matching process. An empty bin means a zero value for frequency. There are four ones in the bins of (*p2*, *q3*), (*p2*, *q4*), (*p4*, *q3*) and (*p5*, *q4*). They can be identified as being falsely matched neighbour spot pairs because their frequencies are relatively low.

Matched pairs by frequencies are determined after the accumulation of frequencies is finished. First, spot pair (*p_5_*, *q_5_*) is chosen as a matched pair because it has the highest frequency of 12. The spots *p_5_* and *q_5_* cannot be chosen again afterwards in other spot pairs because the spot-matching process is a one-to-one correspondence. Then, the second highest frequency of 8 is chosen and spot pair (*p4*, *q4*) is determined. In this way, all of the spot pairs can be determined one by one. Spot pairs (*p_1_*, *q_1_*), (*p_2_*, *q_2_*), (*p_3_*, *q_3_*), (*p_4_*, *q_4_*), (*p_5_*, *q_5_*) and (*p_6_*, *q_6_*) are determined as a result.

These results are more reliable than the results by TPNS because the frequency reflects the times where a spot pair is matched and confirmed by other neighbour spots. If a spot pair has a frequency of 12, it has been proven to be correct matching 12 times by neighbour spots. The frequency for a spot pair is equal to the number of neighbour spots in the case of successful matching. There are some cases when the frequency is lower than the number of neighbour spots, indicating that some matches failed for different reasons. The probability for correct matching can be evaluated by Equation (5). This is very informative because it shows to what extent the spot pairs could be considered correct.
(5) 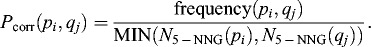



In the previous papers, the spot-matching problem was just a ‘yes’ or ‘no’ question and there were many cases of false positive matches in the results. Researchers had to double check the results from matching algorithms manually and had no indication which and how many pairs needed to be checked. In the proposed method, each spot pair has a probability for correct matching, which narrows down the number of pairs that have to be checked manually, starting from the lowest possibilities. If the spot pair with the lowest matching possibility proves to be a false positive case in the manual check, the checking range can be expanded towards a slightly higher possibility. Otherwise, the results of the spot matching can be convincingly considered to be correct.

## Results and discussion

### Experiment

**Figure 5.**
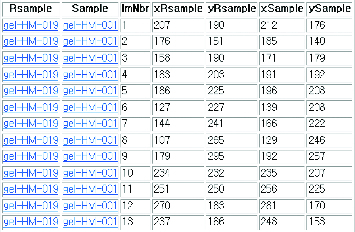
Format of landmark.tbl.

In 2DE, spot detection must precede spot matching. The centroids of spots obtained from the stage of spot detection are very important information for spot detection. The stage of spot detection is omitted for objective evaluation of the spot-matching algorithm. This is done because spot detection is also error-prone and it affects spot matching to a great extent. The data set ‘human leukaemias’ from the website [13] was used. This set has 128 pairs of gels and each gel has approximately 22 manually matched pairs of spots. Information for matching spots between the reference gel and the target gel (as shown in Figure 5) can be downloaded as a text (‘landmark.tbl’).

Rsample and Sample are names used for the reference gel and the target gel, respectively, and ImNbr is a series of matching numbers. xRsample, yRsample, xSample and ySample are central coordinates of spots from the reference gel and the target gel. Two spots on the same line mean they are a matched pair.

Matching information on 128 pairs of gels is originally stored in a single file called ‘landmark.tbl’ altogether. For the purpose of the experiment, the information from the original file is divided into 128 separate files, one file per one pair of gels, respectively. Each gel pair has one-to-one matched pairs and there is no outlier. The same spot numbers are assigned for two spots of matched pairs and matching can be considered right if spots with the same spot number are matched. The programming language Perl was used to implement the proposed algorithm and Python with Turtle graphic library was used to visualize the matching results.

Gel number 34 is selected and the proposed method is demonstrated step by step as an example. Table 1 shows spot-matching information for gel number 34.

**Table 1.** Spot-matching information for gel no. 34.

xRsample	yRsample	xSample	ySample	lmNbr
207	190	197	206	1
176	151	166	161	2
158	190	149	205	3
183	203	177	222	4
186	225	180	244	5
127	227	132	250	6
144	241	144	263	7
107	265	113	287	8
179	295	176	321	9
234	232	221	251	10
251	250	237	265	11
270	183	252	198	12
237	166	221	177	13
295	218	279	235	14
304	285	292	305	15
247	313	239	337	16
198	325	195	351	17
95	350	110	372	18
104	386	120	415	19
156	381	166	412	20
204	460	209	496	21
285	417	289	451	22

**Figure 6.**
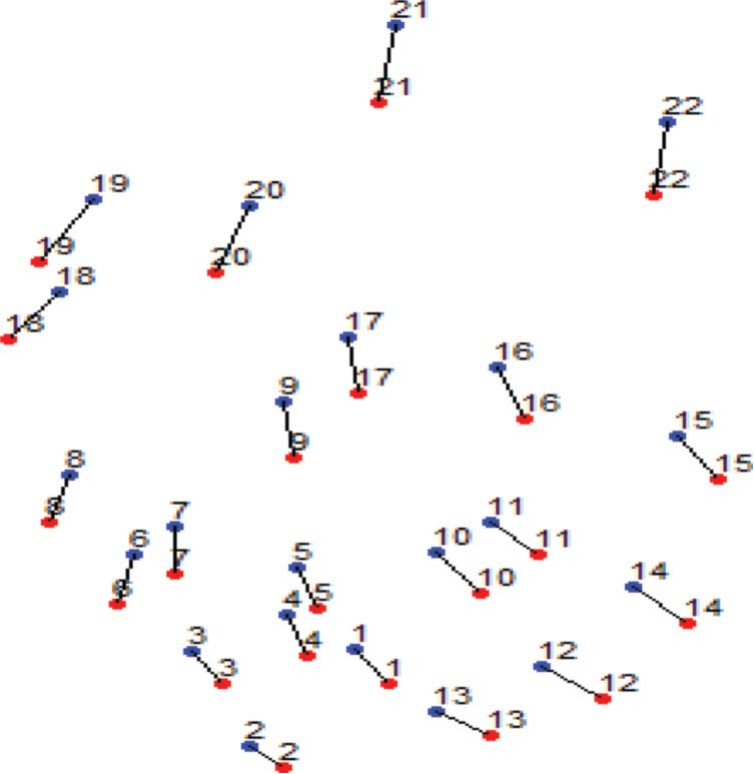
Topological pattern of gel number 34.

Figure 6 shows a visual representation of the results in Table 1. The spots in red are from the reference gel and the ones in blue are from the target gel. Lines between two spots indicate that the two spots linked together are matched spot pairs written on the same line in Table 1. It can be confirmed that the reference gel and the target gel have global distortion and in many cases local distortions as in Figure 6.

**Figure 7.**
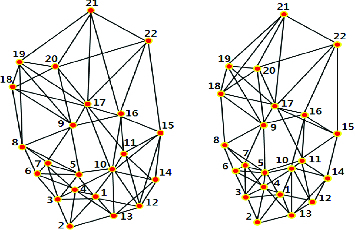
5-NNG graph of reference (left) and target gel (right).

A 5-NNG graph of reference and target gel no. 34 is given in Figure 7. The number of edges for one spot can be more than five, although 5-NNG is applied. The number of edges from one spot is exactly 5 but there are also edges from other spots to it. The number of edges can be more than 5 if all the edges are summed up.

**Figure 8.**
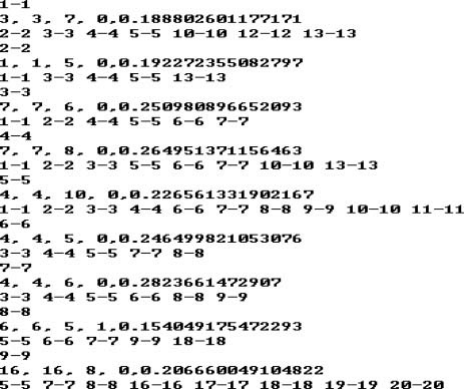
Part of result obtained by TPNS.

Figure 8 demonstrates part of a matching result by TPNS for gel no. 34. In the first three lines, 1–1 means central spot pair from the reference gel and the target gel and the third line shows neighbour spot pairs, 2–2, 3–3, 4–4, 5–5, 10–10, 12–12 and 13–13. They are first of all matched and are then used in matching the central spots.

**Figure 9.**
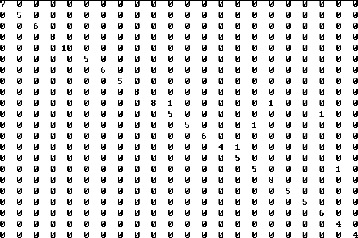
Cumulative frequency matrix of gel no. 34.

The information on neighbour spot pairs is utilized in matching their central spots. A cumulative frequency matrix can be formed with it. Figure 9 shows the cumulative frequency matrix accumulated with the information on the neighbour spot pairs of gel no. 34. The structure of Figure 9 is the same as that of Figure 4. Twenty-two spots from the target gel are on the *X*-axis and 22 spots from the reference gel are on the *Y*-axis; the total number of 484 bins are shown in Figure 9.

Table 2 shows the result where 2761 pairs are correctly matched out of 2763 total number of pairs. A detection rate and matching accuracy of 100% and 99.93%, respectively, were obtained. The detection rate is the total number of detected pairs, including false positive and true positive ones, divided by the total number of 2763 pairs. The matching accuracy is the rate of the number of true positive matches among the detected pairs.

**Table 2.** Result from experiment.

Item	Values
Total number of gel pairs	128
Total number of spot pairs	2763
Number of correctly matched spot pairs	2761
Detection rate	100%
Matching accuracy	99.93%

## Conclusions

A verification method for spot matching in 2D gel electrophoresis images by neighbour spots is proposed. Verification of matched spot pairs is conducted by accumulating occurrences of neighbour spot pairs into a cumulative frequency matrix. Verified and refined information on spot pairs can be obtained and probabilities for correct matching can be evaluated using frequencies and number of neighbour spots for a spot. The proposed method can verify the results of TPNS by comparing them with the results of spot matching produced using cumulative frequency matrix. The researcher can get a hint to what extent they can trust the result obtained by the automated spot-matching algorithm. It presents fairly better results than TPNS. What is more, the proposed method can give probabilities for correct matching of spot pairs and can help researchers decide whether a result can be trustworthy or how many spot pairs they should check manually to accept all the results as true positive ones, TPNS. The proposed method can be used as a complementary tool to TPNS, which might show worse performance in the case of densely populated spots. The proposed method can boost the matching accuracy as well as help researchers verify results from spot matching in less time and with the least possible effort.
